# The FAAH inhibitor URB597 suppresses hippocampal maximal dentate afterdischarges and restores seizure-induced impairment of short and long-term synaptic plasticity

**DOI:** 10.1038/s41598-017-11606-1

**Published:** 2017-09-11

**Authors:** Roberto Colangeli, Massimo Pierucci, Arcangelo Benigno, Giuseppe Campiani, Stefania Butini, Giuseppe Di Giovanni

**Affiliations:** 1 Laboratory of Neurophysiology, Department of Physiology and Biochemistry, Faculty of Medicine and Surgery, University of Malta, Msida, Malta; 2 Department of Experimental Biomedicine and Clinical Neurosciences (BIONEC), Human Physiology Section, University of Palermo, Palermo, Italy; 3 European Research Centre for Drug Discovery and Development (NatSynDrugs) and Department of Biotechnology, Chemistry, and Pharmacy, Università degli Studi di Siena, Siena, Italy; 4 School of Biosciences, Cardiff University, Cardiff, UK

**Keywords:** Hippocampus, Inhibition-excitation balance, Long-term potentiation, Epilepsy

## Abstract

Synthetic cannabinoids and phytocannabinoids have been shown to suppress seizures both in humans and experimental models of epilepsy. However, they generally have a detrimental effect on memory and memory-related processes. Here we compared the effect of the inhibition of the endocannabinoid (eCB) degradation versus synthetic CB agonist on limbic seizures induced by maximal dentate activation (MDA) acute kindling. Moreover, we investigated the dentate gyrus (DG) granule cell reactivity and synaptic plasticity in naïve and in MDA-kindled anaesthetised rats. We found that both the fatty acid amide hydrolase (FAAH) inhibitor URB597 and the synthetic cannabinoid agonist WIN55,212-2 displayed AM251-sensitive anti-seizure effects. WIN55,212-2, dose-dependently (0.5–2 mg/kg, i.p.) impaired short-term plasticity (STP) and long-term potentiation (LTP) at perforant path-DG synapses in naïve rats. Strikingly, URB597 (1 mg/kg, i.p.) was devoid of any deleterious effects in normal conditions, while it prevented seizure-induced alterations of both STP and LTP. Our evidence indicates that boosting the eCB tone rather than general CB1 activation might represent a potential strategy for the development of a new class of drugs for treatment of both seizures and comorbid memory impairments associated with epilepsy.

## Introduction

In temporal lobe epilepsy (TLE), the hippocampus is crucially involved in seizure expression, and abnormality in this structure is associated with memory deficits^1^. People with epilepsy (PWE), especially with TLE, are at significant risk of cognitive impairment associated with seizures^2,3^. The most commonly used antiepileptic drugs (AEDs) are effective in suppressing seizures with marginal therapeutic effect on psychiatric comorbidities such as cognitive alterations associated with epilepsy^4^. In addition, experimental evidence in animal models and PWE shows that antiepileptic treatments can alter *per se* hippocampal functions which often overlap with dysfunctions caused by seizures themselves thereby contributing to further impair learning and memory processes^5,6^. Cannabinoids (CBs) have been suggested in the treatment of several neurological disorders associated with abnormal neuronal excitability and compelling evidence shows that CB compounds might be good candidates for the treatment of different types of epilepsy^7,8^. However, it has been widely reported that exogenous natural and synthetic cannabinoids negatively affect memory processes and alter physiological synaptic plasticity in memory-related brain regions^9–11^. Long term potentiation (LTP) of excitatory transmission is widely accepted as a cellular basis of hippocampus-dependent memory^12,13^ and *in vitro* and *in vivo* studies have demonstrated that exogenous activation of CB1 receptor negatively affects the induction of hippocampal LTP^10,14–16^.

The two major endocannabinoids (eCBs) are anandamide (AEA) and 2-arachydonoil glycerol (2-AG) which suppress synaptic transmission by binding to presynaptic CB1 receptors. eCBs are synthetized and released *on demand* following depolarization-induced intracellular calcium increase and their degradation occurs in a very rapid and accurate fashion^17,18^. AEA is broken down by the enzyme fatty acid amide hydrolase (FAAH)^19^ which keeps AEA levels under constant control to enable a fine tuning of synaptic transmission^20,21^.

However, contrasting evidence exists regarding the role of the eCBs on the induction of LTP. For instance, AEA impaired LTP at CA3-CA1 synapses *in vitro*, through CB1 receptor signalling^22–24^. The FAAH inhibitor URB597^25^ restored the cognitive impairment in chronic cerebral hypoperfusion (CCH) rat model without effecting the normal synaptic plasticity^26^. On the other hand, it has been shown that eCB-mediated depolarization-induced suppression of inhibition (DSI), can facilitate LTP induction in single CA1 neurons activated within an unpotentiated population of neighbouring neurons^27^. As yet, only one study has investigated the role of the AEA at perforant path–dentate granule (PP-DG) synapse *in vivo*; URB597 partially restored age-related decrease in LTP in the DG possibly by modulating microglial activation but was devoid of any activity on LTP in young rats^28^.

AEA together with 2-AG, acts as a key regulator of glutamate and GABA release providing protection against excessive neuronal activity, for example during epileptic seizures^29,30^, and modulates different forms of synaptic plasticity in excitatory and inhibitory neurotransmission in several brain regions^20,31^. Thus, the neuronal activity-dependent action of AEA, which allows selective local tuning of inhibitory and excitatory synapses in hippocampal networks, may represent a good target for suppression of seizures without altering the physiological synaptic homeostasis that hexogen CB1 agonists instead induce.

To test this hypothesis, we examined the antiseizure effect of URB597 in the maximal dentate activation model of limbic seizures in anesthetized rats^32^. Then, we investigated the effect of URB597 on short and long-term plasticity (STP and LTP) at PP-DG cell synapses in normal condition and after repeated seizures. We compared these effects on seizures and plasticity with those induced by the non-selective cannabinoid receptor agonist WIN 55,212-2 (WIN). The involvement of the CB1 receptors was evaluated by blocking URB597 and WIN effects by using the selective CB1 receptor antagonist/inverse agonist AM251.

## Results

### Effects of WIN55,212-2 and URB597 on MDA parameters

**Figure 1:**
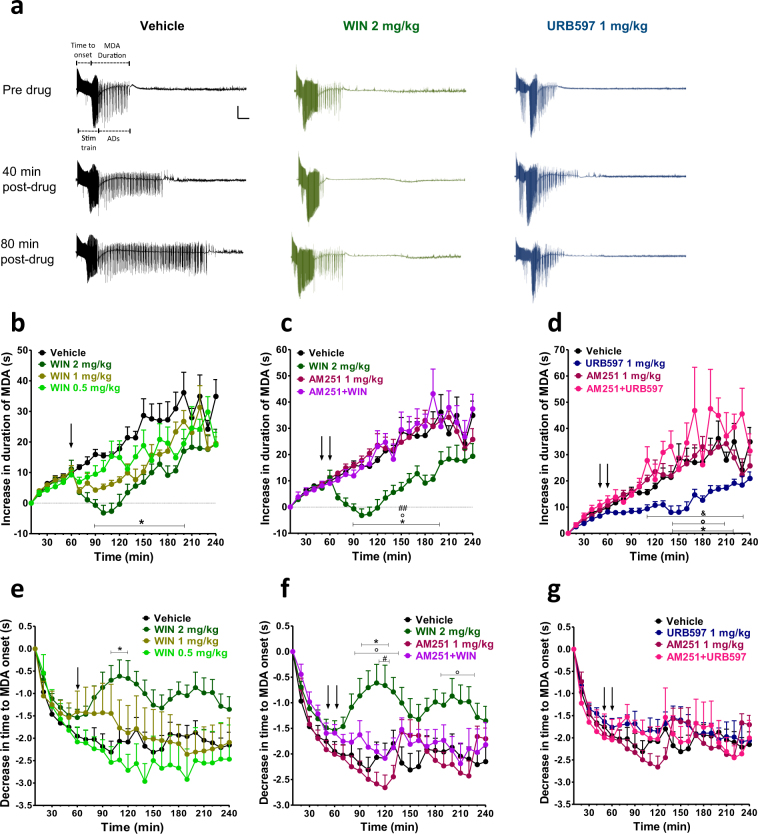
Maximal dentate activation (MDA) after perforant path (PP) stimulation. (**a**) Extracellular field potential recordings from dentate gyrus (DG) of vehicle, WIN55,212-2 (WIN) and URB597 treated rats. Scale bar = 6 mV and 5 s. WIN dose-dependently prevented the progressive increase of MDA duration over time and the progressive increase of the time to MDA onset (**b, e**), while URB597 significantly affected the increase of MDA duration with no effect on the time to MDA onset (**d, g**). Administration of the CB1 receptor antagonist AM251 prevented the effects of WIN and URB597 (**c, d, f** and **g**). The duration and time to onset of MDA were measured for each stimulus train. These values were then normalized, averaged and plotted ( ± SEM) against stimulus number. Drugs were administered i.p. at the arrows. The first arrow refers to AM251 injection or his vehicle, the second arrow refers to either WIN or URB597 administrations or their vehicle. *p < 0.05 vs vehicle; °p < 0.05 vs AM251; ^#^p < 0.05, ^##^p < 0.01 vs AM251 + WIN; ^&^ p < 0.05 vs AM251 + URB597, n = 5–12 per group.

The effects of vehicle, WIN (2 mg/kg i.p.) and URB597 (1 mg/kg i.p.) on MDA at different time points are shown in Fig. 1a. In the vehicle group (n = 9), stimulus trains caused a progressive decrease in the time to onset of MDA over time which reached a steady state around stimulus train 10 and a progressive increase of MDA duration which occurred until the end of the MDA protocol (stimulus trains 24). WIN dose-dependently (0.5–2 mg/kg, i.p.; n = 5 for WIN 0.5 mg/kg, i.p.; n = 5 for WIN 1 mg/kg and n = 10 for WIN 2 mg/kg) reduced both the progressive increase of MDA duration and the progressive decrease of the time of MDA onset (Fig. 1b and **e**; MDA duration: repeated measures ANOVA, time × treatment interaction, F_69,575_ = 2.02; p < 0.0001; time to MDA onset: repeated measures ANOVA, time × treatment interaction, F_69,575_ = 2.60; p < 0.0001). The statistical significance was obtained only with a dose of 2 mg/kg of WIN as revealed by Bonferroni’s post-hoc test (p < 0.05 WIN 2 mg/kg, i.p., vs vehicle; from 90 min to 200 min after the start of the MDA for MDA duration and from 100 min to 120 min for MDA onset; Fig. 1b and e, respectively).

As shown in Fig. 1c and f, the effect of WIN (2 mg/kg, i.p.) was completely prevented by the pre-administration of the CB1 receptor antagonist AM251 (1 mg/kg; AM251 alone n = 8 and AM251 + WIN n = 6). Repeated measures ANOVA showed a significant time × treatment interaction effect for MDA duration (F_69,667_ = 2.69; p < 0.0001) and time to MDA onset (F_69,667_ = 3.42; p < 0.0001). Post-hoc tests revealed a significant difference between WIN 2 mg/kg and AM251 + WIN for both MDA duration (P < 0.01) and time to MDA onset (P < 0.05). As compared to vehicle group, AM251 1 mg/kg, i.p. had no effect on MDA parameters when injected alone.

URB597 (1 mg/kg, i.p.; n = 12) administration significantly prevented the progressive increase of MDA duration and URB597 effect was blocked by pre-administration of AM251 (1 mg/kg, i.p.; repeated measures ANOVA, time × treatment effect, F_69,690_ = 2.62; p < 0.0001, Fig. 1d; URB597 vs vehicle, p < 0.05 from 140 min to 220 min after the start of the MDA; AM251 + URB597 vs URB597, p < 0.05, Fig. 1d). Conversely, the time to onset was not significantly altered by either URB597, or AM251 + URB597 administrations (neither treatment effect F_3,30_ = 0.36; p = 0.7794, nor time × treatment interaction effect F_69,690_ = 1.31; p = 0.053; Fig. 1g).

### Effects of WIN55,212-2 and URB597 on LTP

**Figure 2:**
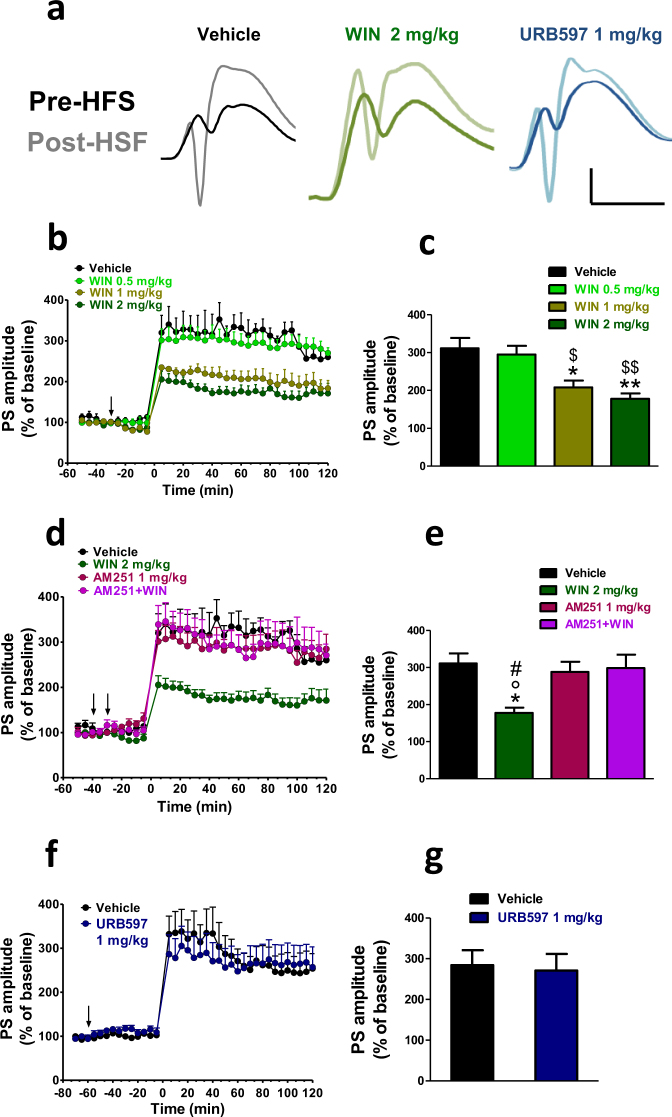
WIN but not URB597 impaired the induction of the LTP in naïve rats. In (**a**) representative DG field potentials in vehicle, WIN (2 mg/kg) and URB597 (1 mg/kg) treated rats at the baseline (dark traces) and 10 min after HFS (light traces). Scale bar = 3 mV and 5 ms. (**b**,**d**,**f**). Time course changes of the average normalized (against baseline) population spike (PS) amplitude before and after HSF-induced LTP. Drugs were administered i.p. at the arrows. The first arrow refers to AM251 injection or its vehicle, while the second arrow refers to either WIN or URB597 administrations or their vehicle. Each point represents the mean ± S.E.M. expressed as a percentage of baseline values. (**c**,**e**,**g)**. A combined plot of the averages of the PS amplitude after HFS. Each group is presented as the mean ± SEM. One-way ANOVA followed by Bonferroni’s post-hoc test; *p < 0.05, **p < 0.01 vs vehicle group; ^$$^p < 0.01, ^$^p < 0.05 vs WIN 0.5 mg/kg; ^#^p < 0.05. vs AM251 + WIN 2 mg/kg; °p < 0.05 vs AM251.

Figure 2a shows the population spike (PS) amplitude recorded before and after high-frequency stimulation (HFS) in rats treated with vehicle, WIN (2 mg/kg, i.p.) and URB597 (1 mg/kg, i.p.). In vehicle treated rats (n = 4 for each different experimental condition), HFS induced a potentiation of the PS amplitude which lasted for the entire recording time (120 min).

WIN (0.5, 1, and 2 mg/kg, i.p.; n = 5–6 per dose), injected 30 min before the HFS, dose dependently impaired LTP (1-way ANOVA; F_3,19_ = 10.49; p = 0.0005) in naïve rats. Statistical significance with Bonferroni’s post-hoc test was obtained with both the dose of 2 mg/kg and 1 mg/kg as shown in Fig. 2b and c (WIN 2 mg/kg, i.p. vs vehicle; p < 0.001 and WIN 1 mg/kg, i.p. vs vehicle p < 0.05).

Pre-administration of the CB1 antagonist AM251 (1 mg/kg, i.p.) prevented the impairment effect of WIN (2 mg/kg, i.p.) on LTP as shown in Fig. 2d and e (n = 5; 1-way ANOVA; F_3,20_ = 5.489; p = 0.0080), while it was devoid of any effect when administered on its own (1 mg/kg, i.p.; n = 6; p = n.s.).

Conversely, URB597 (1 mg/kg, i.p.; n = 5) administered 60 min before the HFS, did not affect LTP, as the PS amplitudes recorded after the HFS were no different to those of the vehicle group (student’s t-test; t_7_ = 0.2352; p = 0.8208; Fig. 2f and g).

### Effects of WIN55,212–2 and URB597 on post-MDA LTP

**Figure 3:**
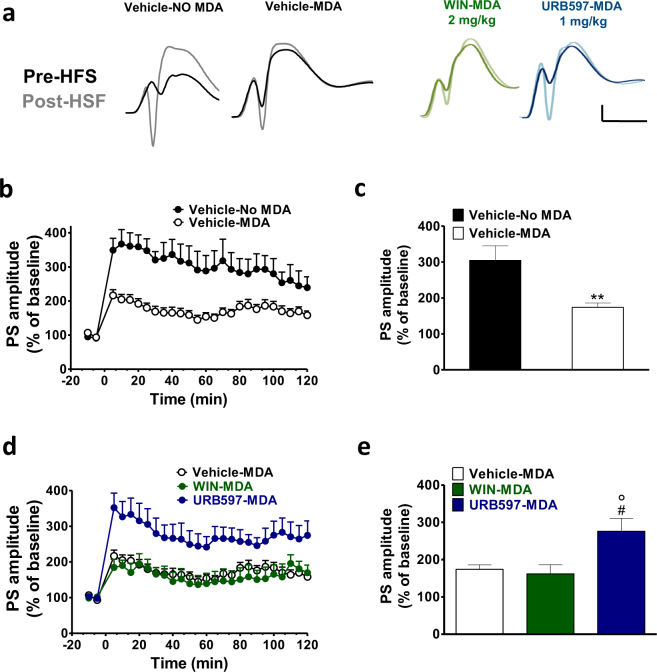
URB597 but not WIN reverted the seizure-induced LTP impairment in rats subjected to MDA acute kindling. In (**a**) representative field potentials at the baseline (dark traces) and 5 min after HFS (light traces) recorded in naïve rat (vehicle) and in rats subjected to MDA kindling in presence of vehicle (Vehicle-MDA), WIN (2 mg/kg) (WIN-MDA) and URB597 (1 mg/kg) (URB597-MDA). Scale bar = 3 mV and 5 ms. Dot plots (means ± SEM expressed as a percentage of baseline values) of relative population spike (PS) amplitudes from 10 min before to 120 min after HFS in control and MDA group (**b**) and in Vehicle post-MDA, WIN (2 mg/kg) post-MDA and URB597 (1 mg/kg) post-MDA groups (**d**). HFS was delivered at time point ‘0.’ (**c**,**e**). A combined plot of the averages of the PS amplitude after HFS. Each group is presented as the mean ± SEM. One-way ANOVA followed by Bonferroni’s post-hoc test; **p < 0.01 vs vehicle group; ^#^p < 0.05 vs vehicle-MDA group; °p < 0.05. vs WIN-MDA group.

To test the effect of repetitive seizures on LTP, HFS was delivered to the PP-DG synapses after the MDA procedure at the end of the 4 h stimulation protocol. 10 min baseline recordings were performed at the end of the MDA, then HFS was delivered and PSs were recorded for 120 min. Figure 3a shows the PS amplitude before and after HFS delivered in animals just treated with vehicle (Vehicle-NO MDA), and in animal subjected to MDA and treated with vehicle (Vehicle-MDA), WIN (2 mg/kg, i.p.; WIN-MDA) and URB597 (1 mg/kg, i.p.; URB597-MDA) where the injection was performed at the 6^th^ train stimulation of the MDA protocol.

Rats subjected to the MDA paradigm (Vehicle-MDA; n = 8) displayed a significantly lower HFS-induced potentiation when compared to the level of LTP of the control group (Vehicle-No MDA n = 4) (student’s t-test; t_10_ = 4.082 p < 0.0022; Fig. 3b and c).

Rats subjected to MDA paradigm and treated with WIN (WIN-MDA; n = 5) showed no difference in the magnitude of LTP compared to the vehicle-MDA group (Fig. 3d and e). Conversely, in rats treated with URB597 during MDA (URB-MDA; n = 7) the magnitude of LTP was significantly greater compared to the vehicle-MDA group (1-way ANOVA; F_2,19_ = 6.147; p = 0.0098; p < 0.05 URB597-MDA vs vehicle-MDA and p < 0.05 URB597-MDA vs WIN-MDA; Fig. 3d and e) and not different from vehicle-NO MDA (student’s t-test; t_9_ = 0.5113; p = 0.6214).

### Effects of WIN55,212-2 and URB597 on paired pulse stimulations

**Figure 4:**
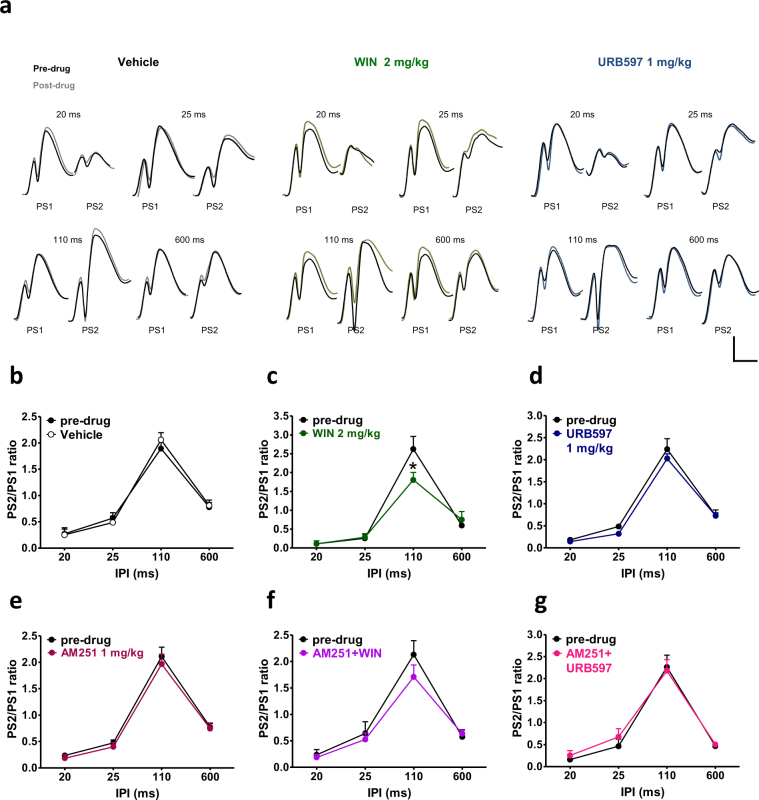
WIN, but not URB597 negatively affected STP in naïve rats. In (**a**) representative field potentials after paired stimulations 20, 25, 110 and 600 ms apart at the pre-drug condition (dark traces) and after drug administration (coloured traces) in vehicle, WIN (2 mg/kg) and URB597 (1 mg/kg) treated rats. Scale bar = 2 mV and 5 ms. Graphs represents the mean ratios at each interpulse interval (IPI) before (pre-drug) and after administration of drugs. Administration of vehicle (**b**), URB597 (**d**), AM251 (**e**) and AM251 + URB597 (**g**) had no effect on STP, whereas WIN selectively impaired paired pulse facilitation (**c**). The effect of WIN was abolished by pre-administration of AM251 (**f**). One-way ANOVA for repeated measures followed by Bonferroni’s post hoc test; *p < 0.05 versus control (pre-drug) group.

Responses of the DG to paired pulse stimulations in control (pre-drug) condition and after the administration of vehicle, WIN (2 mg/kg, i.p.) or URB597 (1 mg/kg, i.p.) are shown in Fig. 4a. In all rats tested, in pre-drug condition, paired identical pulses at different interpulse intervals (IPIs) resulted in PSs depression with short IPIs (20 and 25 ms) followed by facilitation with intermediate IPI (110 ms) and a late inhibition with long IPI (600 ms; Fig. 4a). Injection of vehicle (n = 5) did not alter the paired pulse index as shown in Fig. 4b (repeated measures ANOVA IPIs × vehicle interaction effect, F_3,24_ = 0.32; p = 0.8091). Repeated measures ANOVA showed a significant IPI × WIN 2 mg/kg interaction effect in rats treated with WIN compared to the pre-drug condition (F_3,18_ = 6.45; p = 0.0037; n = 4). Post-hoc analysis revealed a selective decrease of paired-pulse facilitation at 110 ms IPI caused by WIN as compared to the pre-drug condition (p < 0.05; Fig. 4c). Pre-treatment of AM251 (AM251 + WIN; n = 6) completely prevented the effect of WIN 2 mg/kg on paired pulse facilitation as no difference between pre-drug condition and after the co-administration of AM251 + WIN was found (repeated measures ANOVA, no IPI × AM 251 + WIN 2 mg/kg, i.p. interaction effect; F_3,30_ = 1.20; p = 0.3276, and no AM 251 + WIN 2 mg/kg effect; F_1,10_ = 0.60; p = 0.4560; Fig. 4f). AM251 (1 mg/kg, i.p.; n = 6) injected alone had no effect on DG responses to paired pulse stimulations when compared to the vehicle group (IPI × AM 251 1 mg/kg, i.p. interaction effect F_3,30_ = 0.10; p = 0.9578; AM 251 1 mg/kg effect F_1,10_ = 0.78; p = 0.3987; Fig. 4e). Responses of the DG to paired pulse stimulations were not affected by either URB597 (n = 6; IPI × URB597 1 mg/kg, i.p. interaction effect F_3,30_ = 0.37; p = 0.7728; URB597 1 mg/kg, i.p. effect F_1,10_ = 2.09; p = 0.1787; Fig. 4d) or AM251 + URB597 administration (n = 5; IPI × AM251 + URB597 interaction effect F_3,24_ = 0.58; p = 0.6310; M251 + URB597 effect F_1,8_ = 0.11; p = 0.7474; Fig. 4g).

### Effects of WIN55,212-2 and URB597 on post-MDA paired pulse stimulations

**Figure 5:**
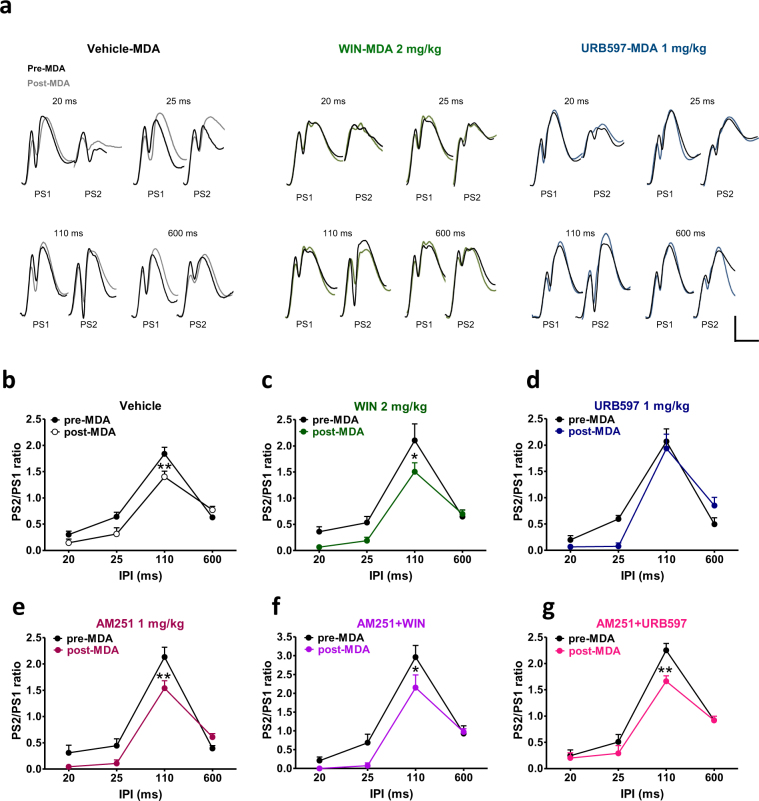
URB597, but not WIN restored paired pulse facilitation in rats subjected to MDA acute kindling. (**a**) Representative DG field potential responses after paired stimulations 20, 25, 110 and 600 ms apart in vehicle, WIN 2 mg/kg, and URB597 1 mg/kg before MDA induction (dark traces) and at the end of 4 hours stimulation at frame 24 (coloured traces). Scale bar = 2 mV and 5 ms. MDA acute kindling impaired paired pulse facilitation in vehicle (**b**), WIN (**c**) and AM251 (**e**) and AM251 + WIN (**f**) groups. Conversely, administration of URB597 was able to prevent the MDA-induced alteration of paired pulse facilitation (**d**) and this effect was blocked by the pre-administration of AM251 (**g**). Drugs were injected one hour after MDA induction at stimulus n. 6. Graphs represents the mean ratios at each interpulse interval before (pre-drug) and after administration of drugs. One-way ANOVA for repeated measures followed by Bonferroni’s post hoc test; *p < 0.05 and **p < 0.01 versus pre-MDA condition.

To test the effect of repetitive seizures on short-term plasticity (STP), paired pulse stimulations were delivered to the PP-DG synapses before (pre-MDA) and after the MDA procedure (post-MDA). Drugs were injected during the MDA protocol at stimulation train 6. Responses of the DG to paired pulse stimulations in pre-MDA (drug free) condition and in post-MDA condition in presence of vehicle, WIN (2 mg/kg, i.p.) and URB597 (1 mg/kg, i.p.) are shown in Fig. 5a.

As shown in Fig. 5b, in the vehicle group (n = 9), repeated measures ANOVA revealed a significant IPI × MDA condition interaction effect (F_3,48_ = 6.73; p = 0.0007). Post-hoc analysis showed that MDA caused a significant selective decrease of paired-pulse facilitation at 110 ms IPI, as compared to the pre-MDA condition (p < 0.01; Fig. 5b). The administration of WIN (n = 5) did not prevent the impairment of MDA on paired pulse facilitation as repeated measures ANOVA showed a significant MDA condition effect (F_1,8_ = 7.93; p = 0.0226) a significant IPI effect (F_3,24_ = 45.76; p = 0.0001) but no interaction between both factors (F_3,24_ = 1.58; p = 0.2201). Post-hoc analysis revealed that, in presence of WIN, post-MDA group showed a reduced paired-pulse facilitation at 110 ms IPI compared to the pre-MDA condition (p < 0.05; Fig. 5c).

No difference in the DG responses to paired pulse stimulations were found between the pre-MDA and the post-MDA condition when rats were administered with URB597 (n = 5; repeated measures ANOVA, neither IPI × MDA condition interaction effect; F_3,24_ = 2.74; p = 0.0652, nor MDA condition effect; F_1,8_ = 0.97; p = 0.3528; Fig. 5d), indicating that URB597 prevented the seizure-induced alteration of paired pulse facilitation.

Repeated measures ANOVA revealed a significant IPI × MDA condition interaction effect in rats treated with AM251 alone (n = 5; F_3,24_ = 4.70; p = 0.0102). Post-hoc analysis showed that, in presence of AM251, MDA induced a selective reduction of paired-pulse facilitation at 110 ms IPI as compared to the pre-MDA condition (p < 0.01; Fig. 5e), indicating that AM251 had no effect on seizure-induced alteration of paired pulse facilitation.

In rats treated with AM251 + WIN (n = 6), repeated measures ANOVA showed a significant MDA condition effect (F_1,10_ = 5.38; p = 0.0429) a significant IPI effect (F_3,30_ = 69.80; p < 0.0001) and no interaction between both factors (F_3,30_ = 2.15; p = 0.1151). Post-hoc analysis revealed that rats subjected to MDA in presence of AM251 + WIN showed reduced paired-pulse facilitation at 110 ms IPI as compared to the pre-MDA condition (p < 0.05; Fig. 5f). URB597 was no longer able to prevent the MDA effect on paired pulse facilitation when administered with AM251 (AM251 + URB597, n = 5; repeated measures ANOVA; IPI × MDA condition interaction effect F_3,24_ = 3.16; p = 0.0430; post-hoc analysis p < 0.01 vs pre-MDA, Fig. 5g).

### Effects of WIN55,212-2 and URB597 on basal synaptic transmission and basal dentate granule cell excitability

To assess basal synaptic transmission and neuronal reactivity of dentate granule cells, field responses were evoked by single PP stimulations delivered at either constant-(time-course) or increasing-current stimuli (100 to 1000 μA; input output (I/O) curve). Compared to the vehicle group (n = 5), neither WIN (2 mg/kg i.p.; n = 5) nor URB597 (1 mg/kg i.p.; n = 5) significantly changed field excitatory post-synaptic potential (fEPSP) slope and PS amplitude during the time of observation (1 h). fEPSP slope; repeated measures ANOVA; neither stimulus strength × treatment interaction, F_28_,_154_ = 0.70; p = 0.8652, nor treatment effect; F_2,11_ = 0.82; p = 0.4649; PS amplitude; repeated measures ANOVA; neither stimulus strength × treatment interaction, F_28,154_ = 0.91; p = 0.5952, nor treatment effect; F_2,11_ = 1.59; p = 0.2465 (data not shown).

**Figure 6:**
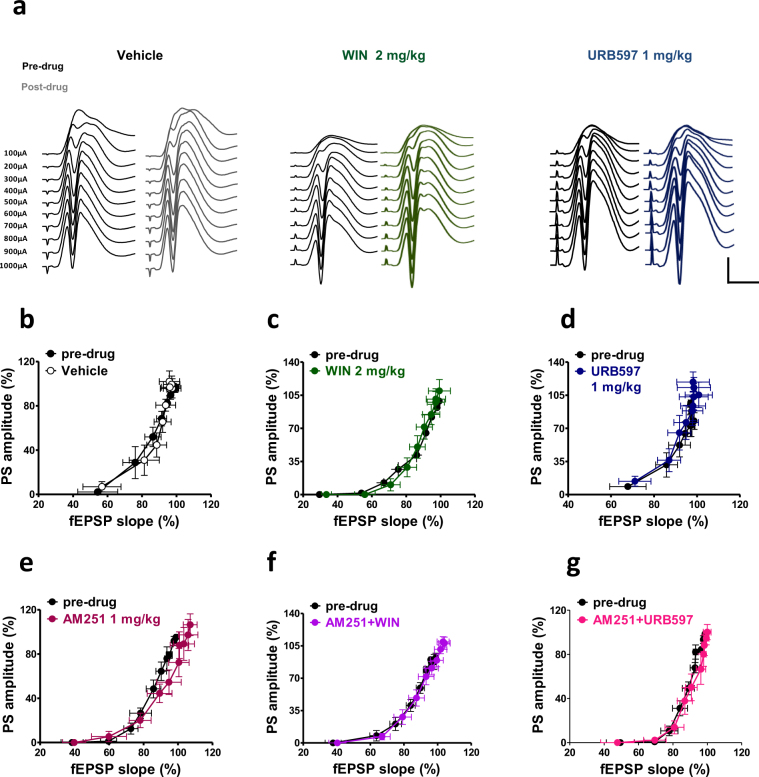
Effects of peripheral administration of cannabinoid compounds on the basal synaptic activity and cellular excitability of dentate granular cells to perforant path (PP) stimulation with increasing stimulation strength. (**a**) Representative DG field potential responses obtained with stimulation intensities ranging from 100 through 1000 μA, recorded in naïve (pre drug) condition and after the administration of vehicle, WIN and URB597. Scale bar = 2 mV and 5 ms. Graphs were obtained by plotting fEPSP slope against PS amplitude for each stimulus strength in pre-drug condition and after drug administration. WIN, URB597 and AM251 did not affect the fEPSP slope and PS amplitude recorded in naïve rats (**b**–**g**).

In Fig. 6a field responses evoked by increasing-current stimuli are shown before and after the administration of vehicle, WIN (2 mg/kg i.p.) and URB597 (1 mg/kg i.p.). For the I/O curve, fEPSP slope and PS amplitude were recorded and normalized to the maximum response obtained during the pre-drug recording. Repeated measure ANOVA revealed no statistical differences in the I/O curve for all drugs tested (fEPSP slope; repeated measures ANOVA; neither stimulus strength × WIN interaction, F_9,72_ = 0.61; p = 0.7829, nor WIN effect; F_1,8_ = 0.08; p = 0.7781; PS amplitude; repeated measures ANOVA; neither stimulus strength × WIN interaction, F_9,72_ = 0.57; p = 0.8164, nor WIN effect; F_1,8_ = 0.24; p = 0.6364; fEPSP slope; repeated measures ANOVA; neither stimulus strength × URB597 interaction, F_9,72_ = 0.09; p = 0.9997, nor URB597 effect; F_1,8_ = 0.05; p = 0.8232; PS amplitude; repeated measures ANOVA; neither stimulus strength × URB597 interaction, F_9,72_ = 0.36; p = 0.9504, nor URB597 effect; F_1,8_ = 2.06; p = 0.1889; Fig. 6b–g).

### Effects of WIN55,212-2 and URB597 on post-MDA dentate granule cell excitability

**Figure 7:**
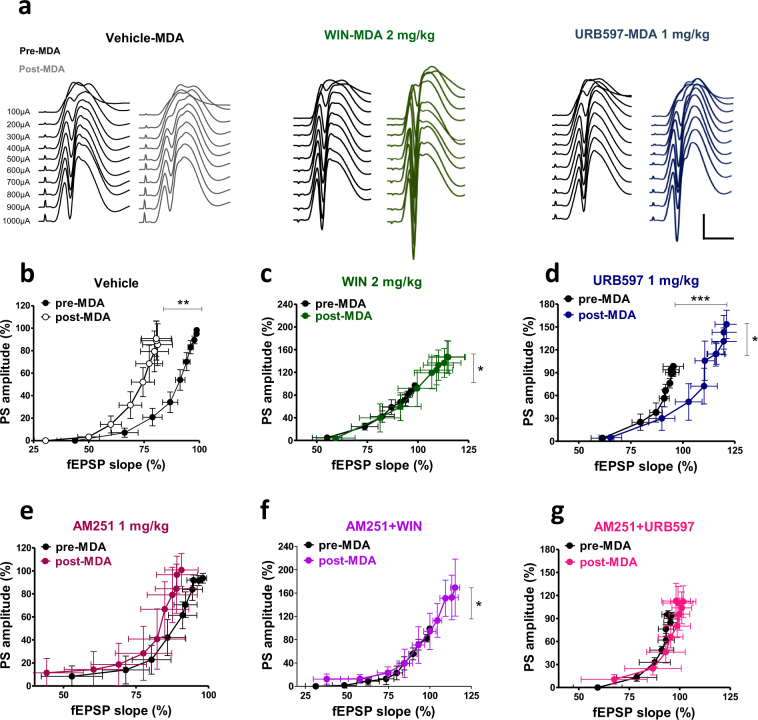
Effects of peripheral administration of cannabinoid compounds on basal synaptic plasticity and cellular excitability assessed in control (pre-MDA, drug free condition) and in post-MDA condition. (**a)** Representative DG field potential responses obtained with stimulation intensities ranging from 100 through 1000 μA, recorded before MDA (drug free condition) and after MDA in presence of vehicle (vehicle-MDA), WIN 2 mg/kg (WIN-MDA) and URB597 1 mg/kg (URB597-MDA). Scale bar = 2 mV and 5 MDA acute kindling caused a significant left-ward shift of the I/O curve in vehicle treated animals (**b**) which was prevented by the administration of WIN and URB597 (**c** and **d**). The left-ward shift of the I/O curve is still present in the AM251-MDA group but no longer significant (**e**). AM251 did not prevent the effect of WIN and only partially prevented the effect of URB597 in the I/O curve (**f** and **g**, respectively). One-way ANOVA for repeated measures followed by Bonferroni post hoc test; *p < 0.05, **p < 0.01 versus pre-MDA condition group.

fEPSP slope and PS amplitude recorded at different stimulus strength in pre-MDA (drug free) condition and in post-MDA condition in presence of vehicle, WIN (2 mg/kg i.p.) and URB597 (1 mg/kgi.p.) are shown in Fig. 7a. In the vehicle group (n = 9), a significant reduction of fEPSP slopes recorded after MDA was found compared to the pre-MDA condition (repeated measures ANOVA; significant MDA condition effect, F_1,16_ = 10.85; p = 0.0046, significant stimulus strength effect, F_9,144_ = 74.60; p < 0.0001, and no significant interaction between both factors, F_9,144_ = 0.15; p = 0.9979), whereas the PS amplitude recorded after MDA was not different compared to the pre-MDA condition (repeated measures ANOVA; neither stimulus strength × MDA condition interaction effect, F_9,144_ = 0.07; p = 0.9999, nor MDA condition effect; F_1,16_ = 0.12; p = 0.7380). This selective effect on fEPSP slopes produced an overall shift to the left of I/O curve (Fig. 7b) indicating an increased excitability of DG cells caused by MDA acute kindling (i.e., firing occurred with less excitatory input). WIN 2 mg/kg, i.p. administration (n = 6) prevented the left-ward shift of the I/O curve caused by MDA, as no differences were found in the fEPSP slope recorded pre and post MDA condition (repeated measures ANOVA; neither stimulus strength × MDA condition interaction effect, F_9,90_ = 0.74; p = 0.6725, nor MDA condition effect; F_1,10_ = 2.75; p = 0.1281). On the other hand, in WIN-MDA group, MDA acute kindling caused a concomitant increase of PS amplitudes recorded at higher current intensities (significant stimulus strength × MDA condition interaction, F_9,90_ = 2.00; p = 0.0485; Fig. 7c). In presence of URB597 (1 mg/kg, i.p.; n = 6) a significant increase of fEPSP slope in post-MDA condition was found with respect to the pre-MDA condition, which caused an overall right-ward shift of the I/O curve (repeated measures ANOVA; significant stimulus strength × MDA condition interaction, F_9,90_ = 3.87; p = 0.0004). Moreover, similar to WIN group, URB597 administration caused an augmentation of the PS amplitude at higher current intensities in the post-MDA condition (repeated measures ANOVA; significant stimulus strength × MDA condition interaction, F_9,90_ = 2.51; p = 0.0.130: Fig. 7d).

The MDA-induced left-ward shift of the I/O curve present in the vehicle-MDA group was no longer significant in presence of AM251 when injected alone (fEPSP slope; stimulus strength × MDA condition interaction effect, F_9,90_ = 0.15; p = 0.9981; MDA condition effect F_1,10_ = 0.90; p = 0.3642; PS amplitude; stimulus strength × MDA condition interaction effect, F_9,90_ = 0.71; p = 0.7025; MDA condition effect, F_1,10_ = 1.05; p = 0.8632; Fig. 7e). In addition, AM251 did not alter WIN effects on the I/O curve (AM251 + WIN, n = 5: fEPSP slope; repeated measures ANOVA, significant MDA condition effect, F_1,8_ = 6.97; p = 0.0297, and stimulus strength effect F_9,72_ = 185.27; p < 0.0001, but not stimulus strength × MDA condition interaction effect, F_9,72_ = 0.77; p = 0.6452. PS amplitude; stimulus strength × MDA condition interaction effect, F_9,72_ = 2.05; p = 0.0456; Fig. 7f), while URB597-induced rightward shift of the I/O and increase in PS amplitude in MDA kindled rats were no longer significant when AM251 was pre-administrated (AM251 + URB597, n = 5: fEPSP slope; stimulus strength × MDA condition interaction, F_9,72_ = 0.05; p = 1.0; MDA condition F_1,8_ = 0.92; p = 0.3647; PS amplitude; repeated measures ANOVA; stimulus strength × MDA condition interaction, F_9,72_ = 0.15; p = 0.9975; MDA condition F_1,8_ = 1.36; p = 0.2779; Fig. 7g).

## Discussion

Firstly, our data show that the synthetic CB agonist and eCBs have a potent antiepileptic effect mediated by CB1 receptors, further confirming compelling experimental and human evidence^7,8^. Secondly, they show that WIN, apart from its antiepileptic effect, dose-dependently altered paired pulse facilitation and impaired the induction of LTP at the PP-DG synapses when tested in normal rats. Conversely, in our conditions, the FAAH inhibitor URB597 did not impair either short- or long-term DG synaptic plasticity at a dose that showed antiseizure effect. Finally, URB597, but not WIN, prevented seizure-induced alterations of short-term synaptic plasticity and restored the capability of the DG granule cells to perform LTP in rats subjected to MDA rapid kindling.

Within the DG, the axon terminals of mossy fibers, PP inputs, mossy cells, and back-projecting CA3 pyramidal cells form an excitatory recurrent network which is suggested to have an important role in learning and memory processes; moreover, excessive activation of glutamatergic transmission within this circuit may lead to epileptiform seizures^33,34^. Furthermore, several types of GABAergic interneurons provide inhibitory control over granule cell activity via feedback and feedforward inhibition^35^. Cannabinoids act mainly at CB1 receptors expressed on both inhibitory and excitatory axon terminals of hippocampal formation, in particular, the DG has one of the highest levels of CB1 receptor expression in the brain^36,37^. Furthermore, degradative enzymes for eCBs are present throughout the DG^38^. Both direct CB1 receptor agonists and eCB degradation inhibitors are effective in controlling seizures in different models of epilepsy^29,30,39,40^, although proepileptic effects have been also reported^41,42^. Here, by using the MDA model of limbic seizures, in which repeated elicitation of PP stimulation in anaesthetised rats produces a progressive increase in afterdischarge duration of DG granule cells^43,44^, we found that both WIN and URB597 prevented the elongation of the MDA duration through a CB1 receptor-dependent mechanism, since this effect was reversed by administration of AM251. Furthermore, WIN, but not URB597, reduced the progressive decrease of the time to MDA onset. The latency to MDA onset can be used as an indicator of seizure threshold (epileptogenesis) while its duration can be used as a measure of seizure activity^32^. Our results suggest that WIN acts on both epileptogenesis and ictogenesis, while URB597 only reduces seizure activity.

Consistent with our findings, it has been reported that WIN significantly delays limbic kindling acquisition in both rats and mice^45,46^ while URB597 does not affect the progression of kindled seizures in the amygdala kindling in mice^45^.

**Figure 8:**
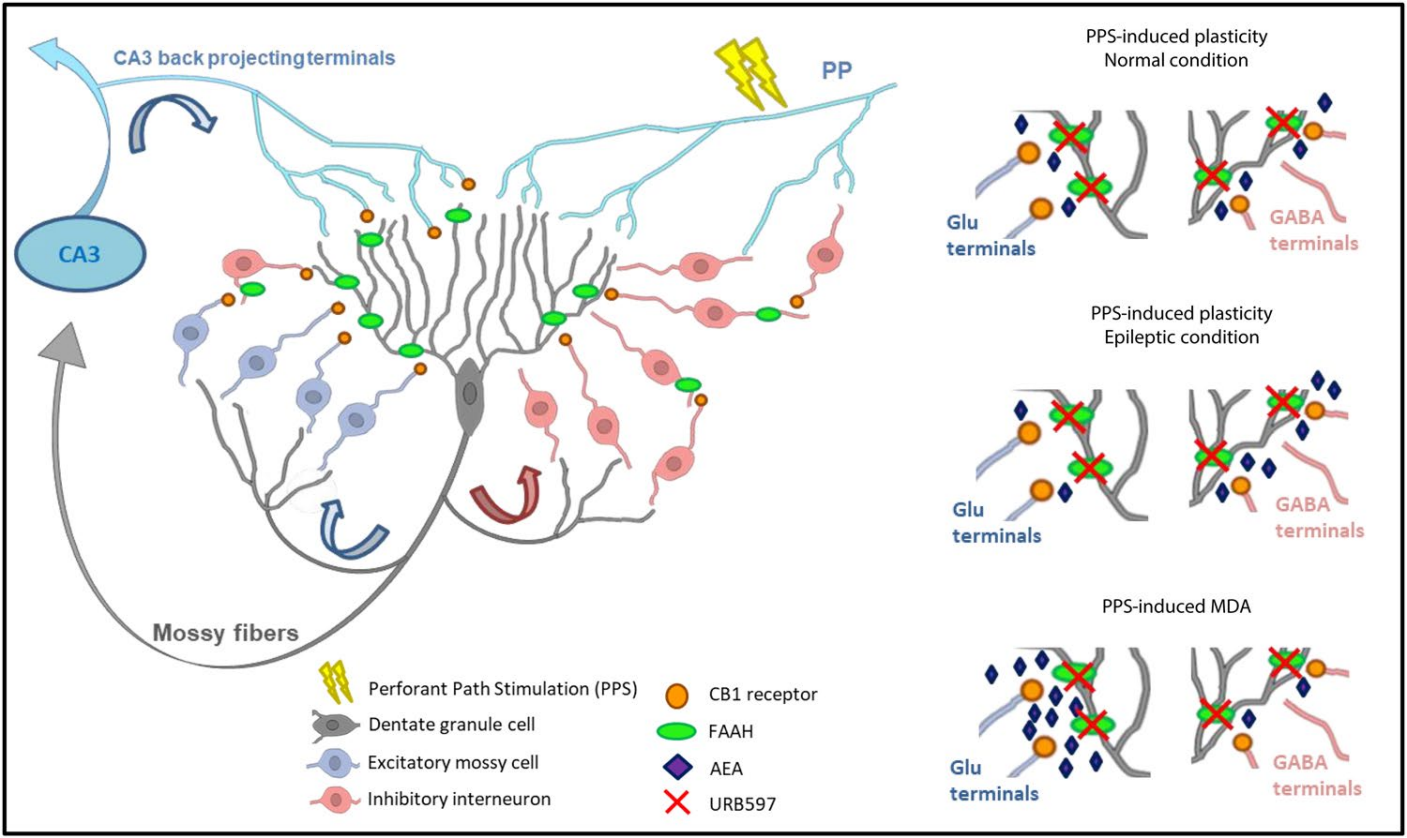
Schematic representation of CB1 receptor and FAAH in the dentate gyrus circuit: Both postsynaptic FAAH and presynaptic CB1 receptor are widely expressed and anatomically associated within the DG except for the PP-DG synapses and mossy fibers that do not express CB1 receptors. On the other hand, CB1 receptor is expressed in the excitatory mossy cell terminals projecting to granule cells and in the CA3 back-projecting terminals to granule cells. CB1 is also heavily expressed in the inhibitory GABAergic terminals, although the expression is restricted to a particular subpopulation of GABAergic interneurons (CCK^+^ interneurons)^36,38^. During PPS (perforant path stimulation)-inducing either synaptic plasticity or MDA, these recurrent excitatory and inhibitory circuits are recruited and, a widespread non-selective activation of CB1 receptor within these circuits, such as after WIN administration, leads to an overall decrease of glutamate release which affects both MDA and synaptic plasticity occurrence. Conversely, AEA release is activity and regional dependent, and a fine tuning of GABA and glutamate transmissions depends on the spatio-temporal biosynthesis and release of AEA might occur in basal condition and during PPF-inducing synaptic plasticity which may further shift towards GABA inhibition when synaptic plasticity is induced in the epileptic circuit. When synaptic activation is strongly and persistently enhanced as during PPS-induced MDA, AEA effect may instead be prominent at glutamatergic synapses. Thus, inhibition of AEA degradation may increase the capability of the AEA to dampen excessive excitability without compromising its temporal and spatial specificity.

Although activation of CB1 receptor causes a decrease in both glutamatergic and GABAergic transmission, there is typically a net reduction in neuronal excitability caused by application of CB1 receptor agonists, likely due to CB1 receptor-mediated presynaptic inhibition of glutamate release^30^. Indeed, several findings have suggested that reduction of glutamate release induced by activation of CB1 receptor expressed at excitatory terminals within the hippocampus plays an important protective role against excessive neuronal activity and epilepsy^29,30^. In our model of limbic epilepsy, the antiseizure effect of WIN may be ascribed to the direct activation of CB1 receptor on glutamatergic terminals, while URB597, by increasing the endogenous concentration of AEA, may potentiate AEA protective effect through a CB1 receptor–mediated inhibition of glutamatergic transmission (Fig. 8).

Interestingly, AM251 at the dose used in this study (1 mg/kg, i.p.) alone did not affect either the MDA elongation or its onset suggesting that PP electrical stimulation that elicits MDA does not induce enough of an increase in eCBs to antiepileptic levels. Our results are consistent with previous findings showing a lack of effect of AM251 in a modified MDA protocol in rats^47^. Therefore, the fact that URB597 is capable of blocking the elongation of the MDA could be due to the increase of the AEA hippocampal concentration to an efficacy level caused by FAAH inhibition.

Indeed, AEA levels are found to rapidly increase in the hippocampus during the ictal event^29^ and reduce in the cerebrospinal fluid of PWE^48^ suggesting that AEA contributes to epileptic seizure termination. Contrasting evidence exists about 2-AG, the other major eCB, that does not increase during kainic acid-induced seizure^29^ and in the cerebrospinal fluid of people with TLE no significant difference in 2-AG levels versus control patients are found ^48^. On the other hand, 2-AG levels are significantly increased 15 min after pilocarpine-induced status epilepticus^49^. Moreover, it is known that 2-AG is responsible for both transient and long-lasting synaptic inhibition in the hippocampus, functioning as a circuit breaker during excessive presynaptic activity^50^ and augmentation of 2-AG levels suppresses excitatory input within the DG contributing to decreased granule cell excitability and seizure occurrence in an acute model of PP kindling^51^. Since it has been reported that FAAH inhibition may affect 2-AG signalling in the striatum and periaqueductal grey^52^ it is possible that the antiepileptic effect of URB597 might be, at least in part, mediated by the modulation of 2-AG signalling. However, it has been shown that acute administration of FAAH inhibitors, including URB597, does not affect 2-AG levels and 2-AG retrograde signalling in the hippocampus^53^.

Previously it has been shown that postsynaptic-depolarization-dependent eCB release from DG cells potently and transiently suppressed glutamatergic inputs from mossy cell but not from PP terminals^54^. Here, by examining the effect of WIN and URB597 on DG granule cell basal synaptic transmission and excitability, we found that neither WIN nor URB597 affected DG cell response to constant-(time-course) or increasing-current stimuli (I/O) delivered to the PP. These findings suggest that the CB1 receptors do not directly modulate PP-DG synapses and the antiseizure effect of WIN and URB597 might occur through the activation of CB1 receptor expressed presumably in mossy cell terminals and/or in CA3 back-projecting terminals once the circuit is hyperactive.

Glutamatergic transmission plays a crucial role in the experimental induction of LTP and a decrease in glutamate release taking place during HFS may prevent or attenuate LTP, and may affect learning and memory^12,13^. There is a large body of evidence reporting the impairing effect of exogenous cannabinoids on the induction of hippocampal LTP^10,14–16^. In line with this evidence, we found that the dose of WIN capable of reducing epileptiform activity also dramatically impaired the induction of LTP through a CB1 receptor-dependent mechanism.

Furthermore, our results showed that WIN, consistent with previous findings *in vitro*^55^, selectively decreased paired pulse facilitation at the PP-DG synapses. LTP and paired pulse facilitation reflect a disinhibition mechanism within the DG circuit which results in enhanced synchronization of spiking activity in the population of granule cells^56,57^. Our findings further suggest that WIN alters synaptic plasticity by dampening activated recurrent network within the DG.

Interestingly, the dose of URB597 that displayed an antiseizure effect did not affect either the induction of LTP or the response to paired pulse in naïve rats. One possible explanation regarding the discrepancies between URB597 and WIN on synaptic plasticity may be that PP stimulation-induced LTP and STP might not significantly increase AEA levels in the DG as occurs during ictal activity (Fig. 8). Indeed, the action of eCBs is both spatially and temporally tightly regulated and FAAH inhibition enhances AEA action only when and where it is synthesised and released^58^. Conversely, a previous study reported enhanced LTP following HFS delivered to the PP and a reduced paired pulse ratio in CB1 receptor knock out (KO) mice^59^, suggesting an involvement of eCBs in the modulation of synaptic plasticity. However, in our condition 1 mg/kg, i.p. of AM251 administered alone did not affect either LTP or paired-pulse response, therefore the alteration of synaptic plasticity in CB1 receptors KO mice might reflect long-term developmental adaptations to chronic absence of CB1 receptors, rather than to the lack of CB1 receptor activation during LTP induction or paired pulse response. Another possible explanation of the URB597 lack of effect on synaptic plasticity may be due to a co-activation of an excitatory mechanism capable of balancing inhibitory AEA effects.

2-AG is known to mediate major forms of CB1 receptor-dependent, short and long-term depression of both excitatory and inhibitory transmission in the hippocampus^17^. Nevertheless, within the DG, 2-AG preferentially affects glutamatergic transmission since diacyl-glycerol lipase enzyme (DAGL) responsible for the synthesis of 2-AG accumulates in the synapses close to glutamatergic terminals and with much less extent at the inhibitory synapses^37^ and DAGL-KO mice show enhanced paired pulse ratio and increased LTP magnitude at the PP-DG synapses^51^. Therefore, in light of this evidence, it is unlikely that FAAH inhibition significantly affects 2-AG signalling in the DG.

The AEA increased levels induced by URB597, in addition to the CB1 receptor, may activate other targets, including TRPV1 receptors. TRPV1 receptors activation increase both excitability^60^, and the induction of LTP at the CA3-CA1 synapses^61^. However, the TRPV1 seems to play an opposite role in the DG compared to CA3-CA1 synapse. Castillo’s group^62^ elegantly demonstrated that at the PP-DG synapse, exogenous activation of TRPV1 receptors in reality reduces excitatory synaptic transmission and both genetic ablation and pharmacological blockade of TRPV1 receptor increase the magnitude of LTP of PP-DG synapses^62^. Therefore, it is unlikely that within the DG, AEA-mediated TRPV1 activation may compensate the CB1 receptor-mediated inhibition of synaptic plasticity.

Strikingly, URB597 during seizures did instead affect synaptic plasticity at the PP-DG synapses when induced after MDA. This different URB597 effect is likely due to the changes induced by paroxysmal discharges, on the induction of LTP, with concomitant reduction of short-term facilitation and increase in intrinsic granule cell reactivity (our observation and^63–67^). Noteworthy, our finding showed that URB597 administered during seizures, differently to CB1 receptor activation by exogenous cannabinoids, was capable of preventing these seizure-induced alterations of synaptic plasticity restoring it to normal levels. This surprising effect on synaptic plasticity in the epileptic PP-DG circuit performed by URB597 might be ascribed to the capability of FAAH enzyme to dynamically modulate AEA action on GABA transmission in the hippocampus (Fig. 8). Indeed, it has been shown that an increased FAAH activity in the hippocampus is associated with a reduced inhibitory control of AEA on GABAergic synapses, which leads to an increased GABA activity^20^. It is possible that, in the epileptic circuit, seizure-induced potentiation of inhibitory synapses, which occurs during interictal periods to counteract hypexcitability^68^, might also mask LTP, thus, inhibition of FAAH and increase AEA onto GABAergic terminals might induce a disinhibitory effect restoring normal LTP.

Several studies reported that FAAH inhibitors produce CB1 receptor-independent molecular, cellular, and functional protection during excitotoxicity and restores synaptic function after brain damage^69,70^. Moreover, it has been shown that chronic URB597 treatment did not alter the basal PP-DG LTP but attenuated the age-related deficit in LTP *in vivo*^28^ and improved chronic cerebral hypoperfusion-induced impairment of both spatial memory and LTP in CA3-CA1 *in vitro*^71^ and these effects might be only partially mediated by CB1 receptors. In addition, our post-MDA I/O results, in acute kindled animals, show that URB597enhaces the threshold of the DG cell excitability at lower currents (input) and potentiate DG cell synchronization (output) for higher current intensities which are only partially reversed by CB1 antagonism, further suggesting a possible involvement of a CB1-independendent action of URB597 in the epileptic circuit within the DG.

In conclusion, URB597, likely boosting AEA tone, dampens abnormal neuronal hyperactivity, does not alter physiological neuronal activity required during synaptic plasticity and prevents seizure-induced alterations of STP and LTP at the PP-DG synapses in epileptic animals. Nevertheless, due to the high complexity of the eCB system in heath and disease state, further work is needed to investigate the underlying mechanism by which URB597 achieves these positive effects. Our findings suggest that inhibiting the FAAH enzyme with contextual increase of AEA rather than general CB1 receptor activation might represent a potential and novel strategy for the development of a new class of drugs for the treatment of seizures and the associated comorbid memory alterations.

## Materials and Methods

### Animals

All experimental protocols were approved by the Institutional Animal Use and Care Committee (IAUCC), University of Malta in accordance with the EU Council Directive 86/609/EEC. Every effort was made to reduce the number of animals used and to minimize their pain and suffering. Male Sprague–Dawley rats weighing ~300 g were used.

### Drugs

The synthetic cannabinoid agonist (R)-( + )-[2,3-Dihydro-5-methyl-3-(4-morpholinylmethyl) pyrrolo[1,2,3-de]-1,4-benzoxazin-6-yl]-1-naphthalenylmethanone mesylate (WIN 55,212-2, Tocris bioscience, UK), the synthetic CB1 receptor antagonist, 4-methyl-1-H-pyrasole- 3-carboxamide (AM251; ABCAM, UK), and the FAAH inhibitor URB597 (kindly provided by Prof. G. Campiani and Prof. S. Butini, University of Siena, Italy) were used. All drugs were freshly dissolved in a vehicle (2 ml/kg) made of 5% PEG-400, 5% Tween 80 in saline and intraperitoneally (i.p.) administered.

### Surgery procedure and electrode implantation

Rats were anesthetized with urethane (Sigma-Aldrich Co., Milano, Italy) (1.2 g/kg, i.p.) and positioned in a stereotaxic frame. Body temperature was maintained by a heating pad and a temperature controller unit (Temperature Control Unit HB 101/2, Letica Scientific Instruments). Surgical procedures were performed according to previous studies^43,72^. Briefly, field potentials were recorded from the hilus of the DG of the hippocampus (AP: −4.8 L: 2.5 V: 3.6) by stimulating the PP (AP: −7.9 L: 4.6 V: 3.4). During the surgical procedure, electrodes (bifilar stainless steel wire, CFW, CA, USA) were advanced slowly downward until the optimal depth to record PSs was reached. A NeuroLog amplifier (Digitimer Ltd, high pass: 0.2 Hz, low pass: 5,000 Hz, gain: 200) was used to record field potentials. Using digitally controlled current stimulator (Digitimer Ltd, model DS3), square-wave pulses of 0.2 ms duration were applied, 1 per min. Stimulus intensity was set to evoke 40–50% of the PS maximum amplitude. Responses were digitized by a CED 1401 plus analogue–digital converter (Cambridge Electronic Design Ltd., Cambridge, UK), stored on a computer and averaged offline using Signal 1.9 software. Sampling rate was set to 10 kHz. Location of the recording electrode was histologically verified at the end of the experiments.

### Maximal dentate activation (MDA)

To elicit MDA, 10 s stimulus trains (pulses of 0.3 ms duration, at 20 Hz) were delivered through the stimulating electrode at an initial intensity of 200 μA. If MDA was not elicited, the stimulus intensity was increased in 50 μA steps and redelivered every 2.5 min until MDA was induced. Stimulus trains were delivered every 10 min for 4 h (total of 24 stimulations). For each stimulus train, the duration and time to onset of MDA were measured^43,73,74^. As shown in Fig. 1a, the time to onset represents the time (in s) occurring from the beginning of the stimulus train to the point where PSs appeared with half of the maximal amplitude. The duration of the MDA, calculated in s, was measured from the appearance of the PSs with half of the maximum amplitude to the end of the afterdischarges (ADs). ADs are represented by spontaneous bursts of PSs that appear at the end of the stimulus train. MDA duration and time to MDA onset were normalized by subtracting the duration (in s) obtained with the first stimulus train from the duration obtained with each subsequent stimulus train (Stringer and Lothman, 1990b). Thus, for individual stimulus train after the first, a change in duration was calculated and data from separate animals were compared and averaged. Either WIN or URB597 were injected after stimulus train 6 (1 h). AM251 was injected after stimulus train 5, alone, or in combination with either WIN or URB597.

### Long term potentiation (LTP)

Tetanic high-frequency stimulation (HFS, 200 Hz) was used to induce long-term potentiation (LTP) at PP-DG synapses. The effect of drugs on LTP was tested in a first set of experiments where HFS was delivered 60 min after URB597 administration and 30 min after WIN administration. The pre-treatment time of WIN, was based on information from previous experiments (Naderi *et al*., 2008). The pre-treatment time of URB597 was chosen on the basis of reported peak brain levels of AEA approximately 60 to 240 min after treatment with URB597^25^. AM251 was injected 10 min before either URB597 or WIN administration. 10 min baseline recordings were performed before each drug treatment and LTP was recorded for 120 min after the HFS.

In a second set of experiments LTP was evaluated after MDA protocol. After the last MDA stimulus train (4 hours after the start of MDA), a 10 min baseline of PSs generated by single pulses was recorded, then HFS was applied to evoke LTP. LTP was recorded for 120 min after the HFS. In this experiment, drugs were administered during the MDA. Either WIN or URB597 were injected after 6 stimulus train. AM251 was injected after 5 stimulus train alone or in combination with either WIN or URB597.

In both the experimental procedures, baseline and post-HFS recordings consisted of single pulses delivered every 30 s and then averaged every 5 responses. HFS consisted of 10 trains of 15 pulses at 200 Hz, with 1 s delay between trains using the same pulse parameters as in baseline. Pulse strength was set to evoke 40–50% of the maximum PS amplitude.

### Paired pulse protocols

To test the dentate granule short-term plasticity, paired-pulse stimulations were delivered to the PP with different interpulse intervals (IPI) (20, 25, 110, 600 ms) to compute the paired-pulse index (PPI). PPI was measured between the second PS amplitude and the first. A ratio of 1 reflects no change in the PS amplitudes evoked by both stimuli. A ratio greater than 1 reflects paired-pulse facilitation and a ratio less than 1 reflects depression. Five responses of each different IPI were averaged. A 30 s gap was maintained between the each stimulation.

In a first set of experiments, PPI was assessed in control (pre-drug) condition and after treatment (post-drug). WIN effect was assessed 30 min after its administration, while URB597 effect was evaluated 60 min after its administration. AM251 was injected 10 min before either URB597 or WIN administration.

In a separate set of experiments, the PPI was assessed in control (pre-MDA) condition and after the last MDA stimulus trains (post-MDA). In this experiment, either WIN or URB597 were injected during the MDA after the 6^th^ stimulus train. AM251 was injected after the 5^th^ stimulus train alone or in combination with either WIN or URB597.

### Time course

To test the drug effects on the basal PP-DG synaptic transmission a time course analysis was performed by continuously delivering single pulses to the PP and assessing fEPSP slope and PS amplitude in control (pre-drug, 15 min) condition and after treatment (post-drug, 1 h). Single pulses were delivered every 30 s and then averaged every 5 responses. Pulse strength was set to evoke 40–50% of the maximum PS amplitude.

### Input output curves

To test the dentate basal granule cell excitability, fEPSP slope and PS amplitude were evaluated by single stimulations delivered to the PP at different pulse strengths, ranging from 100 to 1000 μA, to produce input/output curves by plotting fEPSP against PS amplitude for each stimulus strength. Five responses of each different pulse strength were averaged. A 30 s gap was maintained between the sweeps.

In a first set of experiments, the input/output curves were assessed in control (pre-drug) condition and after treatment (post-drug). WIN effect was evaluated 30 min after its administration, while URB597 effect was evaluated 60 min after its administration. AM251 was injected 10 min before either URB597 or WIN administration.

In a separate set of experiments the I/O curves were assessed in control (pre-MDA) condition and after the last MDA stimulus trains (post-MDA). In this experiment, either WIN or URB597 were injected during the MDA after 6 stimulus trains. AM251 was injected after 5 stimulus trains alone or in combination with either WIN or URB597.

### Statistical analysis

Statistical analyses were performed using statistical software package GraphPad Prism4 (San Diego, CA). The data obtained were compared by the ANOVA analysis of variance for repeated measures followed by Bonferroni’s post-hoc multiple comparisons test. The data were expressed as means ± SEM. Statistical significance was determined at the level of p ≤ 0.05.
