# Author Correction: The FAAH inhibitor URB597 suppresses hippocampal maximal dentate afterdischarges and restores seizure-induced impairment of short and long-term synaptic plasticity

**DOI:** 10.1038/s41598-018-23084-0

**Published:** 2018-03-12

**Authors:** Roberto Colangeli, Massimo Pierucci, Arcangelo Benigno, Giuseppe Campiani, Stefania Butini, Giuseppe Di Giovanni

**Affiliations:** 1 Laboratory of Neurophysiology, Department of Physiology and Biochemistry, Faculty of Medicine and Surgery, University of Malta, Msida, Malta; 2 Department of Experimental Biomedicine and Clinical Neurosciences (BIONEC), Human Physiology Section, University of Palermo, Palermo, Italy; 3 European Research Centre for Drug Discovery and Development (NatSynDrugs) and Department of Biotechnology, Chemistry, and Pharmacy, Università degli Studi di Siena, Siena, Italy; 4 School of Biosciences, Cardiff University, Cardiff, UK

Correction to: *Scientific Reports* 10.1038/s41598-017-11606-1, published online 11 September 2017

The original version of this Article contained a typographical error in the spelling of the author Stefania Butini, which was incorrectly given as Sefania Butini. This has now been corrected in the PDF and HTML versions of the Article.

