# Author Correction: The FAAH inhibitor URB597 suppresses hippocampal maximal dentate afterdischarges and restores seizure-induced impairment of short and long-term synaptic plasticity

**DOI:** 10.1038/s41598-018-37392-y

**Published:** 2018-12-14

**Authors:** Roberto Colangeli, Massimo Pierucci, Arcangelo Benigno, Giuseppe Campiani, Stefania Butini, Giuseppe Di Giovanni

**Affiliations:** 1 Laboratory of Neurophysiology, Department of Physiology and Biochemistry, Faculty of Medicine and Surgery, University of Malta, Msida, Malta; 2 Department of Experimental Biomedicine and Clinical Neurosciences (BIONEC), Human Physiology Section, University of Palermo, Palermo, Italy; 3 European Research Centre for Drug Discovery and Development (NatSynDrugs) and Department of Biotechnology, Chemistry, and Pharmacy, Università degli Studi di Siena, Siena, Italy; 4 School of Biosciences, Cardiff University, Cardiff, UK

Correction to: *Scientific Reports* 10.1038/s41598-017-11606-1, published online 11 September 2017

The Acknowledgements section in this Article is incomplete.

“This work was supported by Malta Council of Science and Technology, grant R&I-2013-14 EPILEFREE to GDG. RC was supported by fellowship funded by EPILEFREE.”

should read:

“This work was supported by Malta Council of Science and Technology, grant R&I-2013-14 EPILEFREE to GDG. RC was supported by fellowship funded by EPILEFREE. MP was supported by a Research Officer Fellowship from the Faculty of Medicine and Surgery, University of Malta. The authors acknowledge the practical contribution of doctoral student Ms Maria Vella.”

